# Comparative Transcriptome and Metabolome Analyses Provide New Insights into the Molecular Mechanisms Underlying Taproot Development and Bioactive Compound Biosynthesis in *Ficus hirta* vahl

**DOI:** 10.3390/genes16070784

**Published:** 2025-06-30

**Authors:** Meiqiong Tang, Chunying Liang, Yude Peng, Hong He, Fan Wei, Ying Hu, Yang Lin, Chunfeng Tang, Gang Li, Linxuan Li

**Affiliations:** 1 Guangxi Engineering Research Center of TCM Resource Intelligent Creation, Guangxi Key Laboratory of Medicinal Resources Protection and Genetic Improvement, National Engineering Research Center for Southwest Endangered Medicinal Materials Resources Development, National Center for TCM Inheritance and Innovation, Guangxi Botanical Garden of Medicinal Plants, Nanning 530023, Chinalggxu07@hotmail.com (G.L.); 2 College of Agriculture, Guangxi University, Nanning 530004, China

**Keywords:** *Ficus hirta* vahl, root development, metabolic biosynthesis, transcriptome, metabolome

## Abstract

Background: *F. hirta* vahl is a famous Chinese medicinal plant. The root is the main organ accumulating bioactive compounds, and its development is directly related to the yield and quality of the harvested *F. hirta.* However, the molecular mechanisms underlying the bioactive compound biosynthesis occurring during the root development of *F. hirta* are unknown. Method: Transcriptome and widely targeted metabolome analyses were performed to investigate gene expression and metabolite variation during the development of *F. hirta* taproots. Results: A total of 3792 differentially expressed genes (DEGs) were identified between the one- and three-year-old *F. hirta* taproots; they are related to circadian rhythm–plant, phenylpropanoid biosynthesis, starch and sucrose metabolism, and plant–pathogen interaction pathways. In total, 119 differentially accumulated metabolites (DAMs) were identified between the one- and three-year-old *F. hirta* taproots, including flavonols, phenolic acids, and coumarins compounds. Integrative transcriptome and metabolome analyses revealed a significant correlation between 172 DEGs and 21 DAMs; they were predominantly enriched for processes associated with phenylpropanoid biosynthesis, flavonoid biosynthesis, plant hormone signal transduction, and stilbenoid, diarylheptanoid, and ginerol biosynthesis. In addition, 26 DEGs were identified to be significantly correlated with the DAMs that accumulated in the phenylpropanoid biosynthesis pathway, and these DEGs may be the key genes for the biosynthesis of *F. hirta* active compounds. Conclusions: The phenylpropanoid biosynthesis pathway plays a dual role in both development and bioactive compound synthesis in *F. hirta* taproots. These findings provide a molecular regulatory network in the relationships between *F. hirta* taproot development and the accumulation of secondary metabolites. The identification of candidate genes and pathways provides a genetic resource for quality control and future molecular breeding in *F. hirta*.

## 1. Introduction

*Ficus hirta* vahl, commonly known as ‘Wu-zhi-mao-tao’, is a popular Chinese ethnic herbal medicinal plant from the *Ficus* genus (Moraceae). This species is mainly distributed in Southern China and Southeast Asian countries. Traditionally, *F. hirta* roots are used as the primary medicinal resource to treat spleen deficiency syndrome, night sweats, postpartum agalactia, weakness, and lung- and liver-related diseases [1]. *F. hirta* contains a wide range of natural active compounds, such as coumarins, flavonoids, steroids, phenolic compounds, amino acids, volatile oils, etc. According to modern pharmacological research, these constituents possess multiple therapeutic properties such as antioxidant, antifungal, antitumor, and anti-inflammatory functions and properties, among others [2,3,4]. Coumarins and flavonoids are regarded as the predominant active compounds of *F. hirta*, and they mainly accumulate in its roots. Among them, the content of psoralen, a kind of furanocoumarins, is determined as the main quality evaluation index of *F. hirta* [5,6]. But, so far, the molecular mechanisms of *F. hirta* active compound biosynthesis, especially the synthesis of coumarins, are poorly understood. Numerous studies have shown that coumarins originate from L-phenylalanine, undergo a common precursor pathway to synthesize umbelliferone, and then differentiate into different coumarins classes [7]. Currently, the key enzymes including phenylala-nine ammonia-lyase (PAL), cinnamate 4-hydroxylase (C4H), and 4-coumarate-CoA ligase (4CL) in the upstream steps of coumarins biosynthetic pathways have been elucidated [8], and prenyltransferase (PT) [9,10], marmesin synthase (MS) [11], and psoralen synthase (PS) [12] were identified as the critical enzymes of furanocoumarin biosynthesis, but a few enzymes involved in biosynthetic steps and the information about the detailed pathway leading to furanocoumarin biosynthesis remain unclear [13].

In cultivation, the taproot is the main commercial organ that directly determines the quality and yield of harvested *F. hirta*. However, the growth year can notably affect the levels of active ingredient accumulation in root medicinal crops [14]. For example, the content of most saponins in *Panax notoginseng* roots increased with age, as measured from 1 to 3 years of growth [15]. The content of triterpene in *Codonopsis lanceolata* roots decreased with age [16]. The amount of major medicinal ingredients in 2-, 3-, and 15-year-old *Scutellaria baicalensis* roots showed a “V” type [17]. Currently, the *F. hirta* plants are usually harvested in the third year of planting, when the biomass and main active ingredients of the taproots are considered optimal [18,19]. In conclusion, the content of and changes in medicinal plant active compounds are related to the developmental stages of the plant. Notwithstanding, current research in this field primarily focuses on the changes in active compounds in different developmental stages at the phenotypic level, and there are no systematic studies to explore the mechanism of the effect of growth stage on the changes in their secondary metabolites.

Plant root growth and development are regulated by both intrinsic molecular mechanisms and external environmental factors [20]. Multi-omics integrative analysis has contributed to the large-scale identification of key genes and metabolic pathways involved in regulating root development. Mitsui et al. [21] reported that the genes participating in carbohydrate metabolism demonstrated significantly up-regulated expression during radish (*Raphanus sativus*) root formation and development through genome sequencing and transcriptome sequencing methods; of these, the sucrose synthase 1 (SUS1) might play an important role in carbohydrate metabolism during radish root enlargement. Transcriptome, proteome, and metabolome analysis indicated that the genes involved in lignin, flavonoid, lipids, and amino acids were mainly activated during cassava (*Manihot esculenta*) tuberous root thickening [22]. Unlike staple crops, for which yield is the ultimate breeding goal, the contents of active ingredients is another important indicator that determines the medicinal materials’ quality in medicinal crops. Most of these active compounds are secondary metabolites of plants. Recently, high-throughput and metabolic profiling methods have been applied in combination to elucidate the underlying molecular mechanism of active ingredient accumulation during root development. For example, in *Tetrastigma hemsleyanum*, the lignin-related gene *PER1* was down-regulated during root development, decreasing metabolites involved in flavonoid and phenylpropanoid biosynthesis [23]. However, the relationships between plant root development and secondary metabolite accumulation are poorly understood, especially in medicinal crops.

Traditionally, the harvest time of *F. hirta* has mainly been determined based on the size and appearance of the taproots. Long harvest periods, low yield, and variable quality have been major bottlenecks in the commercial cultivation of *F. hirta* [19]. Elucidating the molecular mechanisms associated with secondary metabolite accumulation during the development of *F. hirta* root is important for improving its yield and quality. Therefore, in this study, transcriptomic and widely targeted metabolomic analyses were performed to compare the differentially expressed gene as well as metabolites in *F. hirta* taproots at different growth stages (one and three years old). These results promote the dissection of mechanisms underlying taproot development and active ingredient accumulation in *F. hirta* and provide foundational data for shortening its cultivation duration and improving its yield and quality.

## 2. Materials and Methods

### 2.1. Plant Materials

One- and three-year-old *F. hirta* plants used in this study were collected in March 2023 from the Jinling planting base (108.08° E, 22.82° N), Guangxi. They shared the same germplasm source and management practices and were planted by Guangxi Zengnian Agriculture and Forestry Development Co., Ltd. in February 2021 and February 2022, respectively. All plants were uprooted, and the thickest root per plant was considered as the taproot for the following experiment. The formal identification of morphological characters was performed by Professor Yude Peng of Guangxi Botanical Garden of Medicinal Plants, and an *F. hirta* voucher specimen (voucher number: DB00849) was kept in Guangxi Key Laboratory of Medicinal Resources Protection, Botanical Garden of Medicinal Plants. The taproot was cut from the stem base junction and cut into slices ~5 mm thick. These slices were immediately transferred to liquid nitrogen and subsequently stored at −80 °C until further experimental use. Three biological replicates were performed from plants of each growth year group, each comprising nine separate plants.

### 2.2. Transcriptomic Analysis

Total RNA was extracted from *F. hirta* taproots using TRIzol^®^ Reagent (Invitrogen, Carlsbad, CA, USA). The libraries were sequenced with an Illumina HiSeq xten/NovaSeq 6000 (San Diego, CA, USA). The raw data were filtered to obtain the clean data; then, these clean data were assembled using Trinity [24]. Gene functions were annotated using Nr, Pfam, Swiss-prot, GO, eggNOG, KEGG, tmhmm, Signal, and CAZy public databases. Gene expression was normalized into FPKM (Fragments Per Kilobase of transcript per Million fragments mapped) using RSEM [25]. DEGs (differentially expressed genes) were screened using DEseq2 [26] as those with fold change ≥ 2 and *p*-value ≤ 0.05.

### 2.3. Widely Targeted Metabolomic Analysis

Metabolite extract and metabolite profiling of *F. hirta* taproots were carried out at HealthCare Metabolic Biotechnology Co., Ltd. (Wuhan, China). In brief, the filtrated extracts were obtained from the freeze-dried root sample with 70% methanol solution, and then they were analyzed by a UPLC-ESI-MS/MS system. A scheduled multiple-reaction monitoring method was conducted to annotate and quantify the metabolites [27]. DAMs (differentially accumulated metabolites) were identified as those with VIP (variable importance in projection) ≥ 1 and fold change ≥ 2 or fold change ≤ 0.5.

### 2.4. Transcriptomic and Metabolomic Integrative Analysis

The Pearson correlation coefficient (R^2^ > 0.9) was used to analyze the correlation between DEGs and DAMs in the comparison of one-year-old vs. three-year-old *F. hirta* taproot groups. The network plot visualization was performed using the Cytoscape software (v 3.7.2).

### 2.5. RT-qPCR Analysis

RT-qPCR (Quantitative Real-Time PCR) was performed using Cham SYBR qPCR master mix (Vazyme Biotech CO., Ltd., China) in the ABI QuanStudio5 RT-PCR amplifier (Thermo Fisher Scientific, Waltham, MA, USA) with three biological replicates. The RT-qPCR primers were designed with Primer 5 and are listed in Supplementary Table S1. The *actin* gene was used as the endogenous control and the gene’s relative expression level was calculated with 2^−ΔΔCT^ method.

## 3. Results

### 3.1. Transcriptome Analysis of F. hirta Taproots

In this study, nine cDNA libraries (one- and three-year-old *F. hirta* taproots) were prepared and analyzed. A total of 297 million clean reads were filtered. The Q20 and Q30 reached over 97% and 93%, respectively, with the average GC content of 46.73% (Table 1). Sequence raw data were submitted to the SRA database (PRJNA1037593). The final assembly of *F. hirta* had 65,552 transcripts and 46,312 unigenes. Among them, 14,775 (31.90%), 13,779 (29.75%), 11,582 (25.01%), 11,290 (24.38%), 9590 (20.70%), 5522 (11.92%), 2354 (5.08%), and 1315 (2.84%) unigenes were annotated in the Nr, Pfam, Swiss-Prot, GO, eggNOG, KEGG, Signal, and CAZy databases, respectively.

**Figure 1:**
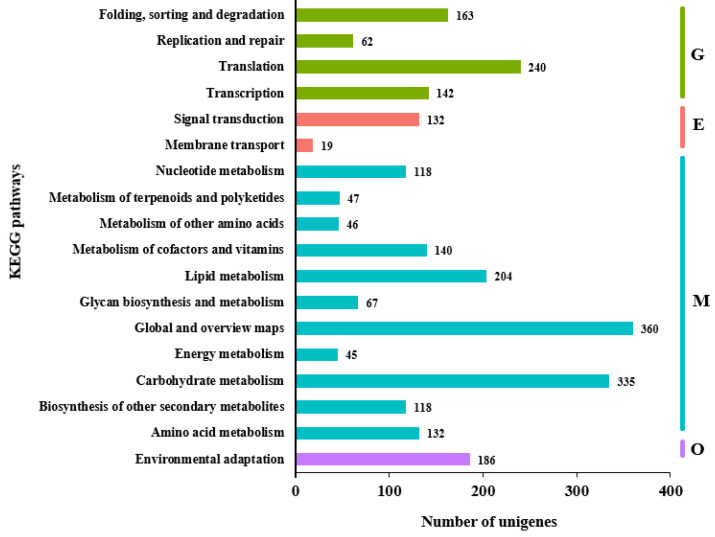
KEGG classification of *F. hirta* unigenes. G, genetic information processing; E, environmental information processing; M, metabolism; O, organismal systems.

These unigenes were assigned into four main KEGG categories: genetic information processing, environmental information processing, metabolism, and organismal systems. Out of the 18 sub-clusters, most KEGG identifiers were categorized in global and overview maps (360 unigenes), followed by carbohydrate metabolism (335 unigenes) and translation (240 unigenes) (Figure 1). Then, the sub-pathways of the significantly enriched metabolism categories were analyzed, and the result showed that the starch and sucrose metabolism pathway (152 unigenes) contained the most unigenes among the carbohydrate metabolism pathways. Regarding the biosynthesis of other secondary metabolite pathways, the phenylpropanoid biosynthesis (107 unigenes) sub-pathway comprised the largest group (Supplementary Table S2).

**Figure 2:**
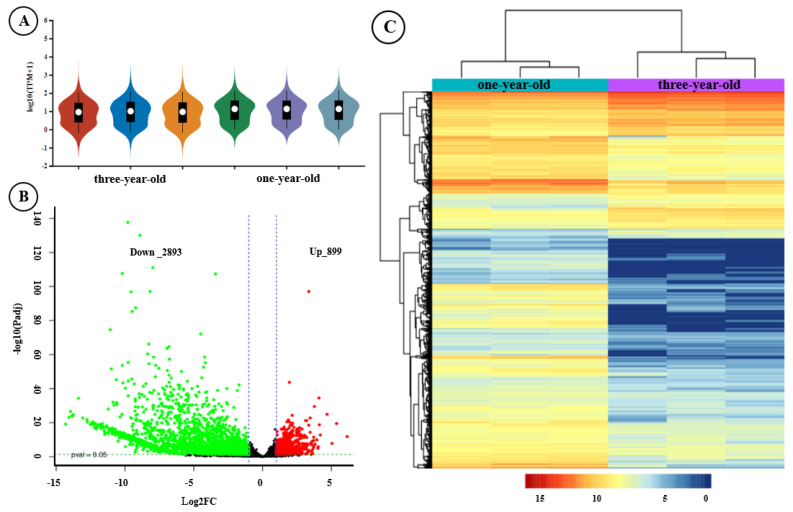
Global gene expression profiling and cluster analysis of DEGs. (**A**) Violin plot of global gene expression distribution. (**B**) Volcano map of DEGs. (**C**) Heat map of DEGs.

Subsequently, the differential transcriptional profiles of *F. hirta* taproots from the one- and three-year-old taproot groups were identified. The gene transcript abundance of all samples was evaluated by assessing the FPKM distribution through the violin plot, which revealed a similar trend and concentrated range of values (Figure 2A). A total of 3792 DEGs were obtained between the two different growth year groups. Compared with the genes expressed in one-year-old *F. hirta* taproots, 899 DEGs were up-regulated, and 2893 DEGs were down-regulated in three-year-old *F. hirta* taproots (Figure 2B, Supplementary Table S3). In addition, the DEG hierarchical clustering was analyzed with DEGs, as shown in Figure 2C; DEG clusters with significant differential expression patterns between the two groups are highlighted on the heat map. These results suggested that one- and three-year-old *F.hirta* taproot gene expression profiles exhibited significant differential expression patterns.

**Figure 3:**
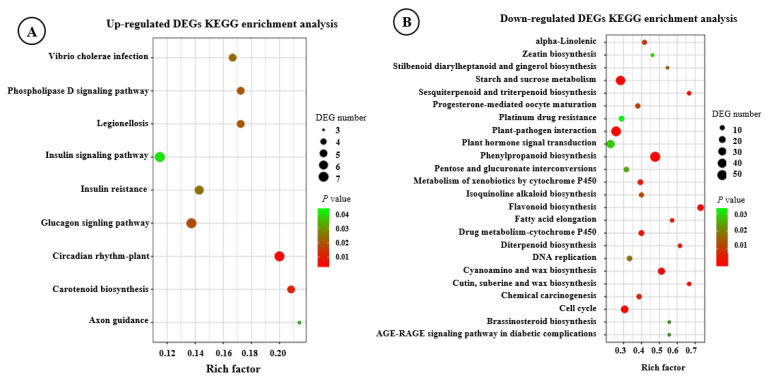
KEGG enrichment analysis of DEGs. (**A**) Up-regulated DEGs enriched KEGG pathway. (**B**) Down-regulated DEGs enriched KEGG pathway.

KEGG pathway enrichment analysis revealed that most DEGs were predominantly enriched in metabolic biosynthesis and signaling pathways. Up-regulated DEGs could be significantly mapped to nine pathways (*p* < 0.05), of which three pathways were involved in the insulin signaling pathway. The circadian rhythm–plant and insulin signaling pathways were enriched with the most DEGs (Supplementary Table S4, Figure 3A). A total of 24 significant KEGG categories were identified that were enriched with down-regulated DEGs. Among them, phenylpropanoid biosynthesis, starch and sucrose metabolism, and plant–pathogen interaction pathways were predominantly enriched (Supplementary Table S4, Figure 3B). The above results indicated that these pathways may be closely associated with the development of *F. hirta* taproots.

### 3.2. Metabolome Analysis of F. hirta Taproots

The secondary metabolites of *F. hirta* taproots from plants of different ages were studied based on a widely targeted metabolomics technique. A total of 388 metabolites were identified, including 89 phenolic acids, 39 alkaloids, 38 organic acids, 33 flavonols, 25 flavones, 24 lignans, 22 coumarins, 14 flavanols, 13 flavonoid carbonosides, 11 flavanones, 11 terpenoids, 11 isoflavones, 9 vitamins, 8 proanthocyanidins, 6 chalcones, 3 anthocyanins, and 32 other compounds (Supplementary Table S5).

**Figure 4:**
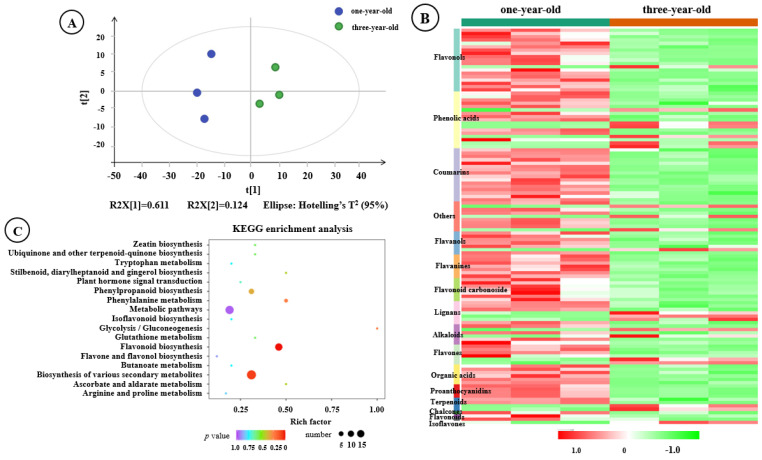
Analysis of DAMs between one- and three-year-old *F. hirta* taproots. (**A**) Principal component analysis of the two groups based on the volatile metabolic profiles. (**B**) A heat map visualization of DAMs. (**C**) KEGG enrichment analysis of DAMs.

PCA revealed the variation in the metabolic phenotypes of *F. hirta* taproots with different ages. PC1 and PC2 explained 61.1% and 12.4% of the total variance, respectively, and all samples in the score plots were within the 95% Hotelling’s T-squared ellipse, with a considerable separation observed between one- and three-year-old taproots groups (Figure 4A). Among 388 metabolites, there were 119 DAMs. Most of the DAMs were flavonols, phenolic acids, and coumarins compounds (Figure 4B, Supplementary Table S6). Of these DAMs, 21 were identified in higher concentrations in the three-year-old taproot group compared to the one-year-old taproot group. By contrast, 98 metabolites in the three-year-old taproot group were significantly down-regulated compared with the one-year-old taproots (Supplementary Table S6).

KEGG enrichment analysis was performed to explore the potential metabolic pathways that were differently regulated in the taproots of *F. hirta* plants of different ages. In total, 18 enriched KEGG pathways were identified among the DAMs (Supplementary Table S7). Based on both *p*-value and pathway rich factors, we identified that the top five enriched metabolic pathways were flavonoid biosynthesis, biosynthesis of secondary metabolites, glycolysis/gluconeogenesis, phenylalanine metabolism, and phenylpropanoid biosynthesis (Figure 4C).

### 3.3. Integrated Transcriptome and Metabolome Analysis

**Figure 5:**
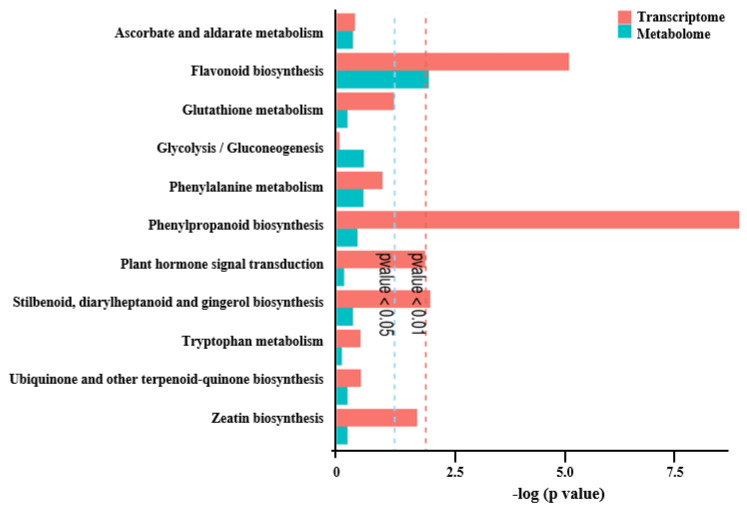
KEGG enrichment analysis of correlated DEGs and DAMs between one- and three-year-old *F. hirta* taproots.

An integrated analysis of both transcriptome and metabolome data was carried out to explore the key genes and metabolic pathways that potentially contribute to *F. hirta* taproot development and metabolite composition. The enrichment analysis of KEGG indicated that a total of 172 DEGs had strong correlation coefficient values (R^2^ > 0.9) with 21 DAMs enriched in 11 pathways; they were associated with phenylpropanoid biosynthesis, flavonoid biosynthesis, plant hormone signal transduction, and stilbenoid, diarylheptanoid, and ginerol biosynthesis (Figure 5, Supplementary Table S8). Notably, the phenylpropanoid biosynthesis pathway exhibited the strongest correlations; therefore, we focus on phenylpropanoid biosynthesis pathway for further analysis.

**Figure 6:**
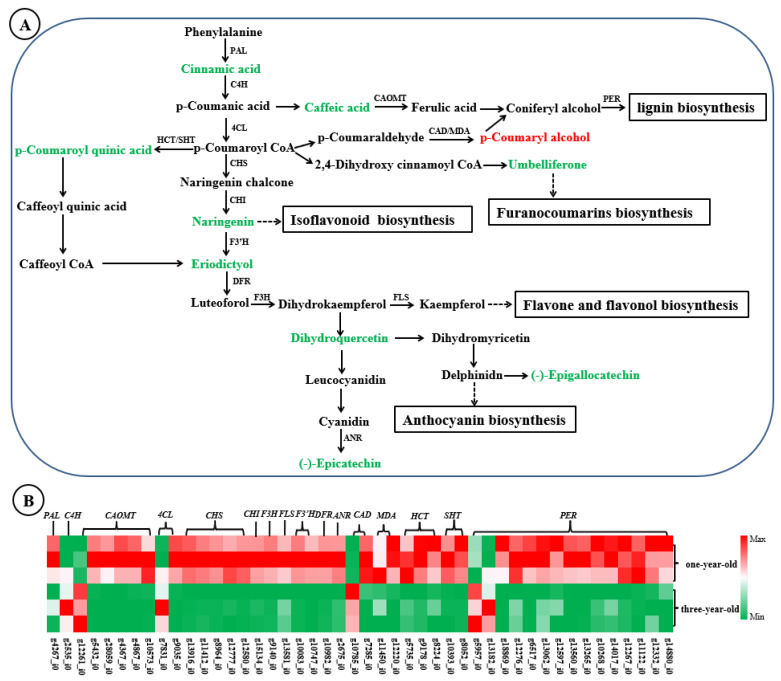
Significant enrichment DEGs and DAMs related to phenylpropanoid biosynthesis pathways in *F. hirta* taproot. (**A**) The pathway of phenylpropanoid biosynthesis. (**B**) Expression profile of DEGs/DAMs related to phenylpropanoid metabolism. Red/green words represent DAMs identified from the integrated analysis results of transcriptome and metabolome; red: up-regulated; green: down-regulated; PAL, phenylalanine ammonia lyase; C4H, cinnamic acid 4-hydroxylase; CAOMT, caffeic acid 3-O-methyltransferase; PER, peroxidase; 4CL, 4-coumarate CoA ligase; HCT, shikimate O-hydroxycinnamoyltransferase; SHT, spermidine hydroxycinnamoyl transferase; CAD, cinnamyl alcohol dehydrogenase; MDA, mannitol dehydrogenase; CHI, chalcone isomerase; CHS, chalcone synthase; F3’H, flavonoid 3′-monooxygenase; DFR, dihydroflavonol reductase; F3H, flavanone3-hydroxylase; FLS, flavonol synthase; ANR, anthocyanidin reductase.

Previous studies have reported that secondary metabolites such as flavonoids, lignin, coumarins, and other compounds are produced through different branches of the phenylpropanoid biosynthesis pathway in plants [28]. So, we constructed the main pathway of phenylpropanoid biosynthesis in *F. hirta* by referencing the phenylpropanoid biosynthesis pathway in the KEGG database with the detected DEGs and DAMs. A total of 46 DEGs and 10 DAMs were identified, among which DEGs included *PAL*, *C4H*, *CAOMT*, 4CL, *CHS*, etc., and DAMs included cummamic acid, caffeic acid, naringein, etc.; they were mainly concentrated on the biosynthetic pathways of the precursors of lignin, coumarins, and flavonoid (Figure 6A). Furthermore, the gene expression and metabolite accumulation in the phenylpropanoid biosynthesis pathway between one- and three-year-old taproots were analyzed. The results revealed that all associated DEGs, except for *C4H* (g2532_i0), *CAOMT* (g12261_i0), *4CL* (g7831_i0), *CAD9* (g10785_i0), *PER* (g5957_i0), and *PER4* (g13182_i0) genes, were found to be down-regulated in three-year-old taproots (Figure 6B, Supplementary Table S3). At the same time, the contents of all DMAs declined in three-year-old taproot, except for p-coumaryl alcohol (Figure 6A, Supplementary Table S6). Overall, the contents of metabolites were basically consistent with the expression trends of their upstream genes.

**Figure 7:**
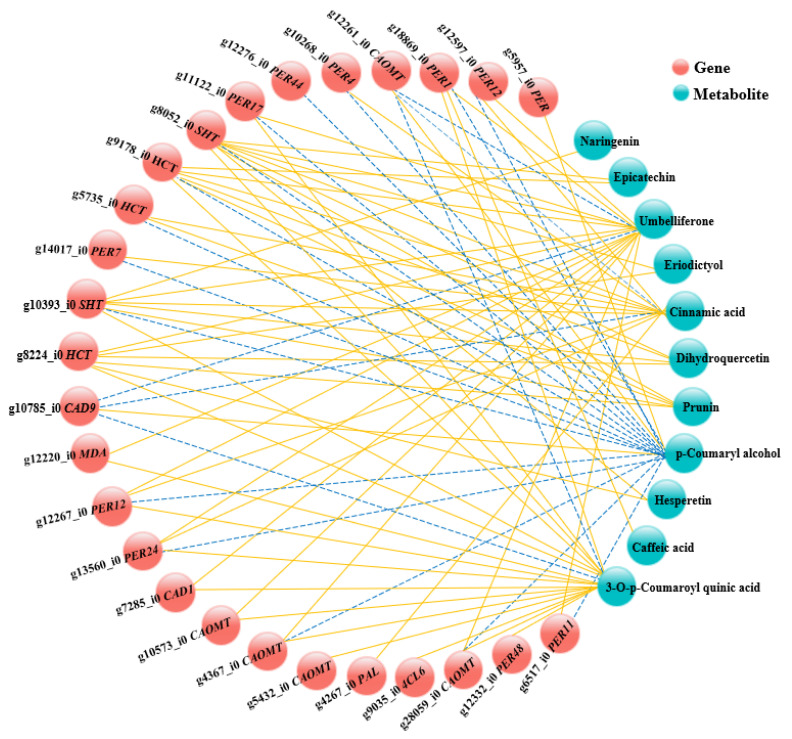
Correlation network between DEGs and DAMs involved in phenylpropanoid biosynthesis. The solid yellow and dashed blue lines represent positive and negative correlation, respectively.

Finally, to further analyze the relationships between the genes and metabolites involved in the phenylpropanoid biosynthesis pathway, a connection network was established to examine the potential regulatory network between DEGs and DAMs (Figure 7, Supplementary Table S9). The results showed that 26 DEGs were significantly correlated with 11 DAMs. Metabolites such as umbelliferone, cinnamic acid, and 3-O-p-coumaroyl quinic acid were highly positively correlated with most DEGs; however, the opposite trend was observed in p-coumaryl alcohol, which showed a negative correlation with most DEGs. In addition, we discovered that, among these DEGs, 11 were *PER* genes, and g8052_i0 *SHT*, g9178_i0 *HCT*, g10393_i0 *SHT*, and g8224_i0 *HCT* were highly positively correlated with most DAMs, indicating that these genes may play an important role in the biosynthesis of phenylpropane compounds in *F. hirta*.

### 3.4. Validation of the Expression Level of DEGs by RT-qPCR

**Figure 8:**
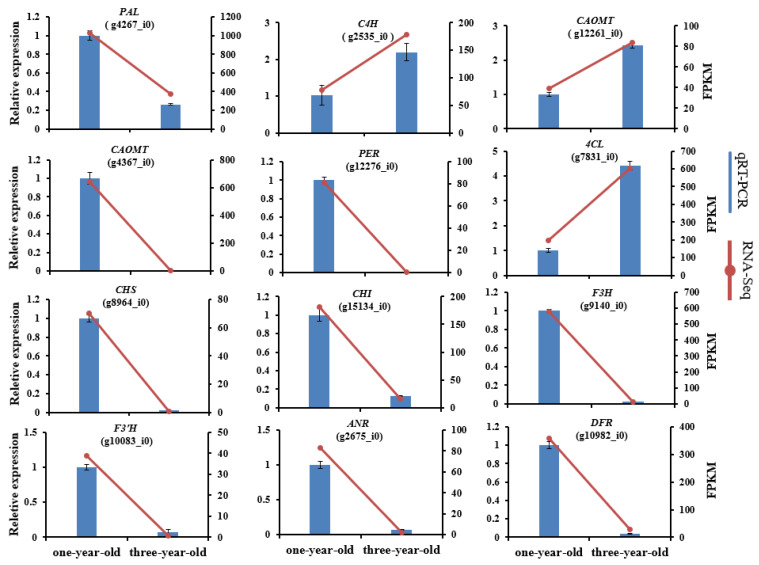
The verification of gene expression trends by RT-qPCR assay. The histogram shows the relative expression data from RT- qPCR; the line chart shows the FPKM value from the RNA-Seq data. Data are mean ± standard deviation (*n* = 3).

To verify the reliability of the transcriptomic data, 12 DEGs involved in the phenylpropanoid biosynthesis pathway were selected randomly for RT-qPCR analysis. All the assessed genes showed similar expression trends between the RNA-Seq data and RT-qPCR results (Figure 8), indicating that the transcriptomic results can accurately reflect the transcriptomic patterns of *F. hirta*.

## 4. Discussion

As an important medicine and food homology plant in China, *F. hirta* has increasingly gained attention for its diverse bioactivities without toxicity. However, most of these studies have focused on chemical composition, pharmacological properties, and agricultural cultivation practices [3,29]; researchers have paid little attention to the genetics of this species. It remains to be determined how active compound accumulation takes place in *F. hirta* developmental as well as the mechanisms by which it occurs. The levels of active compound accumulation in plants showed different spatiotemporal changes with their developmental stage and surrounding environments [30,31]. We investigated the transcriptional changes and secondary metabolite variation in *F. hirta* at different growth years in this study and, thereby, gained insights into the development and accumulation of bioactive compounds in *F. hirta* taproots.

### 4.1. Phenylpropanoid Biosynthesis Pathway Plays a Dual Role Both in Taproot Development and Accumulation of Bioactive Compounds in F. hirta

On the transcriptomic level, thousands of DEGs were identified between one- and three-year-old *F. hirta* taproots, and most DEGs were expressed at higher levels in one-year-old *F. hirta* taproots (Figure 2B, Supplementary Table S3). In the metabolomics analysis, flavonols, phenolic acids, coumarins, and other important secondary metabolites accumulated significantly, and most of them exhibited lower expression in three-year-old *F. hirta* taproots (Figure 4B, Supplementary Table S6). Notably, DEGs and DAMs associated with the phenylpropanoid biosynthesis pathway were identified as being significantly enriched in jointed transcriptomic and metabolomic data (Figure 5). In plants, the phenylpropanoid metabolic pathway is an important secondary metabolism pathway; different branches of the phenylpropanoid biosynthesis pathway are related to the synthesis of different important secondary metabolites, including flavonoids, coumarins, lignin, and others [28,32]. By examining the phenylpropanoid biosynthesis pathway in *F. hirta*, we found that the biosynthesis of phenylpropanoids is a complex process. Phenylalanine is first converted to p-coumanic acid by PAL and C4H, p-coumanic acid is further catalyzed by different enzymes, and then different branches are produced (Figure 6). Umbelliferone, the parent compound of coumarins, is first prenylated in 6- to yield demethylsuberosin and is then catalyzed by a series of enzymes and produces numerous furanocoumarins metabolites such as marmesin, psoralen, bergaptol, 8-methoxypsoralen, and others [7,8,9,10,11,12,13]. The regulatory network further revealed that 16 DEGs were found to be highly correlated with umbelliferone synthesis, including *CAOMT*, *SHT*, *HCT*, *CAD*, *MDA,* and *PER* (Figure 7), which may be closely related to the accumulation of umbelliferone and its downstream derivatives.

Coumarins and flavonoids are plant secondary metabolites. At present, over 7 coumarins and 30 flavonoids have been isolated and identified from *F. hirta* [33]. In earlier research, coumarins were considered to have multiple functions in plants, such as the regulation of oxidative stress and defense biotic and abiotic stress and probably hormonal regulation [7]. Flavonoids have a variety of physiological functions in plants, including growth, development, and reproduction [34,35]. He et al. [36] reported that flavonoid compounds including quercetin, isoquercitrin, and epicatechin are biomarkers related to *Amygdalus pedunculata* growth and development. In our study, a large number of DAMs were identified as coumarins and flavonoids between one- and three-year-old *F. hirta* taproots, and their contents decreased with prolonged root development (Supplementary Table S6). Previous studies have shown that *F. hirta* enters a fast-growing period and has a fast accumulation of active compounds during the first year of cultivation [37,38]. Taken together, we speculate that the levels of accumulation of active compounds in *F. hirta* are determined by two processes: biosynthesis and utilization. The high expression of genes related to the phenylpropanoid metabolic pathway produced many active compounds or the precursors of active compounds, which are necessary for the rapid development of the young tissues in one-year-old *F. hirta* taproots. With prolonged taproot development, active compounds such as coumarins and flavonoids were gradually utilized for maintaining growth, coupled with decreased gene expression at the later stages of development, ultimately leading to a lower content of active compounds in mature *F. hirta* taproots. This result was similar to *P. notoginseng*; phenylpropanoid metabolism is closely related to taproot development and quality [39]. All of the above results suggest that the phenylpropanoid metabolic pathway is important in both development and bioactive compound synthesis in *F. hirta*. taproots.

### 4.2. Effect of Plant Hormone Signal Transduction and Lignin on Taproot Development in F. hirta

Based on transcriptomic and metabolomic data, the plant hormone signal transduction pathway also exhibited a significantly enriched pathway according to the KEGG enrichment analysis, as reflected by the 41 DEGs identified related to this pathway (Figure 5, Supplementary Table S8). Among them, the DEGs were involved in IAA signaling such as indole-3-acetic acid-amido synthetase (g4589_i0, g6226_i0, g6850_i0) and auxin-responsive protein (g15616_i0, g17897_i0, g11333_i0), which were up-regulated in one-year-old *F. hirta* taproots compared to three-year-old *F. hirta* taproots (Supplementary Table S3). Previous studies have demonstrated that IAA concentration reduction and maintenance at a lower concentration are necessary for *P. notoginseng* taproot thickening [40]. Higher auxin increases potato tuberous root formation at the early stage [41]. The expression of genes related to the GA signaling pathway such as gibberellin receptor (g3034_i0) also significantly declined in three-year-old *F. hirta* taproots (Supplementary Table S3). The high content of exogenous GA inhibited the formation of the potato storage organ, while endogenous GA decreased when the stolon tips stared to swell [42]. These results indicate that the plant hormone signal transduction pathway appears to be closely associated with *F. hirta* taproot swelling.

In addition, according to the analysis results of the connection network between DEGs and DAMs, we found umbelliferone to be positively correlated with 6 *PER* genes involved in the phenylpropanoid biosynthesis pathway, including *PER12* (g12597_i0, g12267_i0), *PER1*, *PER17*, *PER24,* and *PER11* (Figure 7, Supplementary Table S9). This result tends to indicate that *PER* may be co-regulated with lignin and coumarin synthesis. However, until now, neither solid evidence for a potential function nor the molecular mode of *PER* have been provided in coumarin biosynthesis. Lignin is an important component of the plant cell wall and participates in cell wall extensibility and cell expansion [43]. In this study, lignin was significantly accumulated in one-year-old *F. hirta* taproots, and the expression of most *PER* genes showed similar changes (Supplementary Table S6, Figure 6B). We speculated that lignin may be essential for the formation of *F. hirta* root at the early stage. For cells with vigorous divisions in the immature *F. hirta* root, high lignin content is beneficial for cell wall extensibility and cell expansion. Congruent with our results, previous studies on *T. hemsleyanum* and *Ipomoea batatas* showed that lignin is needed for the formation of fibrous roots in the early stage [24,44]. So, lignin may play an important role in the development of *F. hirta* taproot.

## 5. Conclusions

In this study, we revealed the effects and mechanisms of growth years on the contents of active compounds in *F. hirta* taproots for the first time. A total of 3792 DEGs and 119 DAMs were identified between the one-year-old taproots and the three-year-old taproots. The expression of *CAOMT*, *SHT*, *HCT*, *CAD*, *MDA,* and *PER* genes were highly correlated with umbelliferone synthesis. Phenylpropanoid biosynthesis was related not only to the development, but also secondary metabolite variation in *F. hirta* taproots. These genes and pathways may be important targets for breeding efforts to improve *F. hirta* yield and quality.

## Figures and Tables

**Table 1:** Statistics of RNA sequencing data.

Samples	Raw Reads	Clean Reads	Clean Bases	Q20 (%)	Q30 (%)	GC Content (%)
one-year-old-1	58,846,288	58,355,866	8,569,908,235	98.05	94.29	46.94
one-year-old-2	52,592,982	52,199,062	7,605,999,070	98.21	94.80	46.56
one-year-old-3	54,274,762	53,825,646	7,875,174,914	98.03	94.31	46.93
three-year-old-1	41,074,490	40,674,184	5,941,654,993	98.00	94.28	46.17
three-year-old-2	48,135,216	47,776,358	6,984,940,329	98.14	94.54	46.90
three-year-old-3	45,302,458	44,878,188	6,566,856,537	97.93	93.99	46.86

## Data Availability

Data are contained within the article or Supplementary Materials.

## Supplementary Materials

The following supporting information can be downloaded at: https://www.mdpi.com/article/10.3390/genes16070784/s1. Table S1: List of primers; Table S2: KEGG analysis of all genes; Table S3: List of DEGs; Table S4: KEGG analysis of up- and down-regulated genes; Table S5: List of all metabolites; Table S6: List of DAMs; Table S7: KEGG analysis of DAMs; Table S8: KEGG analysis of DEGs and DAMs; Table S9: Pearson correlation analysis of DEGs and DAMs.
