# Supplementary material for: Comparative Transcriptome and Metabolome Analyses Provide New Insights into the Molecular Mechanisms Underlying Taproot Development and Bioactive Compound Biosynthesis in Ficus hirta vahl
Source: Genes (Basel). 2025 Jun 30;16(7):784. doi: 10.3390/genes16070784 (PMC12294288; doi:10.3390/genes16070784)
Supplement: Supplementary file 1 [file genes-16-00784-s001.zip › Supplementary Table S1.docx]

Supplementary Table S1. Primers for qRT-PCR

| **Primers for qRT-PCR** | | |
| --- | --- | --- |
| *PAL* (g4267_i0) | Forward | CCTCGCCACCTATCCGTTGATG |
|  | Reverse | TCTTCTCGCTCTCGCCGTTTG |
| *C4H* (g2535_i0) | Forward | TGAGGCTGTATGTTGGAGAAGAAGG |
|  | Reverse | CGACGGAGAGCAGCAGATGG |
| *CAOMT* (g12261_i0) | Forward | GTGCTGCCGATGGTTCTGAAAG |
|  | Reverse | GAGGCGATGTAGGAAGGTGAGAC |
| *CAOMT* (g4367_i0) | Forward | ACGAGGGGTCCCAATGAAACAC |
|  | Reverse | GTGCTTAGTTGCTCTGTGGTTAGTG |
| *CAOMT* (g10573_i0) | Forward | CAAAGGCGTGTGTTCCATAGAGC |
|  | Reverse | TGGGACCCTTGTTGGCGTTG |
| *4CL* (g7831_i0) | Forward | ACAAGGATGGCTACATACAGGTGAC |
|  | Reverse | TGAGAGGATGAGTAAGAAGCAAGGC |
| *CHS* (g8964_i0) | Forward | CGGCTGCACCATCACCAAATAAG |
|  | Reverse | GCTCGTGTCCTCGTCGTCTG |
| *CHI* (g15134_i0) | Forward | CGGCGTTAAACTCCAAGTAAACTCC |
|  | Reverse | GATCGGCGAAGACTCTGTTCCTC |
| *F3H* (g9140_i0) | Forward | TTGACCACATAGGCACCTTCCAC |
|  | Reverse | CGGCTCTTCTTCACATCCTTCATCC |
| *F3’H* (g10083_i0) | Forward | TGATGGTGCTGGCTGGAGTATTC |
|  | Reverse | TCGTCAAGAATTGCGGTCAAGAAC |
| *DFR* (g2675_i0) | Forward | CTGTGTGAACTGGCGATGTAACG |
|  | Reverse | ACTTGGATGACCTCTGCGAAGC |
| *ANR* (g10982_i0) | Forward | CGAAGAGAATTGGAGCGACATTGAG |
|  | Reverse | GAAGGTATCACGGTGACGAGGTC |
| *Actin* (153_i0) | Forward | TTAGACGTTGTGTAGCCAGCA |
|  | Reverse | CTACTTGCGTTGTGATCCGG |
